# Assessment of Nutritional Status and Health Perception among Male Inmates in Israeli Prisons

**DOI:** 10.3390/nu15102255

**Published:** 2023-05-10

**Authors:** Shani Ben Aharon, Ofer Regev, Riki Tesler, Sharon Barak, Yair Shapira, Yossi Weiss, Noa Shtainmetz, Yochanan Vaknin, Liav Goldstein, Kathrine Ben-Zvi, Ruth Birk

**Affiliations:** 1 Nutrition Department, Ariel University, Ariel 40700, Israel; shaniben@gmail.com (S.B.A.);; 2 Health Management Department, Ariel University, Ariel 40700, Israel; ofergev@gmail.com (O.R.); yairsh@ariel.ac.il (Y.S.); yossiw@ariel.ac.il (Y.W.); noa.simplywrite@gmail.com (N.S.); 3 Nursing Department, Ariel University, Ariel 40700, Israel; sharoni.baraki@gmail.com; 4 Department of Pediatric Rehabilitation, The Edmond and Lily Safra Children’s Hospital, The Chaim Sheba Medical Center, Ramat-Gan 52621, Israel; 5 Israel Prison Service, P.O. Box 81, Ramla 72100, Israelkathrineb@ips.gov.il (K.B.-Z.); 6 Chief Medical Officer Office, Israel Prison Service, P.O. Box 81, Ramla 72100, Israel

**Keywords:** inmates, nutrition, subjective health status, well-being, overweight, obesity

## Abstract

The nutritional and health perceptions of inmates are crucial to their overall well-being. However, limited research has been conducted on this topic. This study aimed to assess the nutritional and health perception state of male inmates in eleven prisons in Israel. A cross-sectional study was conducted between February and September 2019 with 176 voluntary participants. Structured questionnaires were used to collect data on socio-demographic characteristics, healthy habits, subjective health status, and prison situation variables. The study found that the prevalence of overweight (40%) and obesity (18.1%) among 18–34-year-old inmates was significantly higher than in the reference Israeli population. Short detention periods (up to one year) predicted less weight gain, while older age predicted poorer health status. Better emotional status significantly predicted better subjective health status among male inmates. There is a need for nutrition interventions to improve the health of inmates. The significant weight gain during incarceration and the associated lower health index and stress highlights the importance of increasing knowledge and promoting a healthier lifestyle in incarceration as early as possible and continuing over time.

## 1. Introduction

Nutrition plays an important role in an individual’s health status, with nutritional deficiencies likely to affect physical and mental health [1]. Given that inmates have a higher incidence of nutrition-related chronic diseases relative to the general population, adequate nutrition can aid in disease prevention and improved quality of life [2]. There are different approaches worldwide regarding prison food and nutrition policies. For example, Scotland and Denmark follow a decentralized approach, giving prison management relative autonomy in food acquisitions and catering [3,4]. Furthermore, in some facilities, prisoners are responsible for managing their food budget and cooking for themselves. The Israeli Prison System (IPS) has a large catering system of supply, transportation, and food budgeting. The IPS’s food branch is also responsible for training staff and supervising food suppliers. Out of 32 prison facilities, about two-thirds have fully functional kitchen services [5]. Due to budget and mixed approaches to managing food, inmates often do not have access to adequate nutrition.

Israeli law, as well as the Eighth Amendment of the U.S. Constitution, protects the rights of inmates to adequate health care. The Israeli Prisons Ordinance indicates that it is the state’s responsibility to provide an adequate confinement environment, including a supply of fresh water and food, to maintain the prisoners’ health. Despite this, studies have shown that prisons often diverge from them [6], leading to inadequate nutrition and health concerns for inmates. Compared to the general population within Israel, inmates are significantly more prone to physical, mental, social, and chronic health problems [7]. Malnutrition in underweight or overweight people is a major cause of mortality and morbidity in prison in developing and developed countries [1]. In developed countries, inmates are gaining more weight, faster, relative to the general population [8]. It has been demonstrated that many inmates sentenced to long incarceration periods gain weight, with obesity rates rising during incarceration [9,10]. Furthermore, obesity co-morbidities, such as diabetes, hypertension, and atherosclerosis are on the rise among inmates [11,12,13,14]. Underweight is also prevalent among inmates, mostly within developing countries [15].

Health inequality is a well-known phenomenon among prison inmates. This was clearly reflected during the COVID-19 pandemic, when high infection rates were documented for inmates and wardens well beyond the rates in the general population [2]. Recognizing the inequality in prisoners’ health led the World Health Organization to initiate projects and frameworks to improve their health [16,17]. Prison inmate health is influenced by both intrinsic and extrinsic factors related (but not limited) to mental, social, physical, and nutritional factors. Characteristics of prisoners vary according to their home country (in high-income countries, the prison population is older, and health issues differ accordingly) [18,19]. Additionally, inmates often come from a deprived environmental background, with limited access to health services and preventative medicine, which a priori establishes a challenging health status [20,21].

A person’s health condition is also affected by their perceptions of well-being. The definition of well-being is complex and comprises several combined areas, including physical, economic, social, emotional, psychological, activity engagement, and work [22]. There is agreement that well-being is a positive outcome that is meaningful for people and for many sectors of society [9]. Since well-being is subjective, it is typically measured with self-reports. The use of self-reported measures is fundamentally different from the use of objective measures.

To the best of our knowledge, there is not sufficient research on the impact of nutrition on inmates’ perceptions of health and well-being. Due to the importance of nutrition as a fundamental pillar of health, the known malnutrition among inmates, and the higher risk of inmates developing nutrition-related chronic diseases, there is a growing and urgent need to address this issue. This study aimed to assess the nutritional and health perception state of male inmates in 11 prisons in Israel.

## 2. Materials and Methods

### 2.1. Participants and Sampling

A cross-sectional study was conducted between 19 February 2019 and 28 September 2019 in 11 prisons in Israel, including three prisons in the northern district, three prisons in the southern district, and five prisons in the central district. Convenience sampling methods were used, and 176 male inmates participated voluntarily in our research. Excluding criteria included inmates who were critically ill and unable to communicate and security prisoners. Data on sociodemographic characteristics, healthy habits, subjective health status, and prison situation variables were collected using a structured questionnaire. The English version of the questionnaire was translated into Hebrew and back-translated to English to check for accuracy. Five data research collectors and one supervisor were employed and given a one-day training session on the procedures and ethics of data collection. The questionnaire was pre-tested on 20 participants (5% of the sample size) in another prison in the region before the study’s data were collected. The internal consistency of the health promotion questionnaire was checked using Cronbach’s alpha; the questionnaire had an alpha value of 0.83, showing a good level of internal consistency.

### 2.2. Questionnaires

The questionnaire was composed of sociodemographic characteristics, health behaviors, participation in health promotion programs, and health status variables.

*Sociodemographic characteristics*. These included participants’ age, gender, marital status, level of education, and country of origin.

*Health behaviors.* These included (1) physical activity; (2) healthy nutrition habits; and (3) unhealthy nutrition habits. Physical activity (PA) was measured using the Exercise and Leisure-Time PA (LTPA) items from the National Health and Nutrition Examination Survey [CDC, 2000]. Frequency and types of leisure time PA (>3 times/week) were assessed. Each activity was coded as “Yes” or “No” and then summed up for the total number of LTPA, which was used to determine whether individuals met activity recommendations. Regarding the duration of each exercise, participants were asked about the length of time they had spent rapid walking, slow walking, running, aerobic training, or weight lifting in the past month. Answers included <150 min (classified as insufficient) or 150 min (classified as sufficient). The reliability of PA was 0.85 [23]. Regarding healthy nutrition habits, participants were asked about the amount of fruits and vegetables (in two separate questions) they eat: a score of 1 meant they tended to eat them once a day or more; a score of 0 meant they ate them less than once a day. The final score ranged between 0 and 2. The reliability of this questionnaire was between 0.85 and 0.90 [24]. Regarding unhealthy nutrition habits, participants were asked about the amount of snacks, fried food, sodas, candy, bread, and butter they consumed on a weekly basis. Answers were given a score of 1 for “rarely” or 0 for all other answers. The final score ranged between 0 and 5. The reliability of this questionnaire was between 0.85 and 0.90 [25].

*Participating in health promotion programs.* Participants were asked about their tendency to participate in yoga, gym, meditation, lectures about smoking, the Vipassana program, and the jangling program. Participation in any program was given a score of 1. The final score ranged between 0 and 7.

Subjective health status (SHS) was measured using the standard question: “How do you evaluate your health generally?” The scale included six levels ranging from 6 = excellent to 1= very bad. The reliability of SHS was between 0.75 and 0.85 [26].

### 2.3. Dependent Variables

Two dependent variables were used: SHS and weight change in prison. SHS was evaluated by asking the following question: “How do you evaluate your health generally?^”^ The scale included six levels ranging from 6 = excellent to 1 = very bad. This variable was also dichotomized to create two categories of SHS: “good to excellent” and “bad and very bad” health.

Weight change in prison was evaluated by asking the question: “Has your weight change since you entered prison?” Answers included weight gain, weight reduction, and no change.

### 2.4. Independent Variables

Several independent variables were used.

*Sociodemographic characteristics.* Sociodemographic variables included age, country of origin, marital status (single, in partnership, married, separated, divorced, or widowed), and education (number of years in school and educational level). Country of origin was dichotomized into “native to the country” and “not native to the country.” Marital status was dichotomized into “in a relationship” (in relationship or married) and “not in a relationship” (single, separated, divorced, or widowed).

*Imprisonment characteristics.* Participants were asked to report on two characteristics related to their imprisonment: years of detention (up to one year, one to five years, at least five years) and the number of family visits to the prison during the previous month (never, once, two to four times, more than four times). The latter was dichotomized into “no visit” (never) and “at least one or more visits per month” (once, two to four times, more than four times).

*Environmental factors.* Three environmental factors were assessed: PA level, eating behavior, and products bought in the prison store. Regarding PA, participants were asked about their current weekly time (minutes) spent in MVPA, including rapid walking, slow walking, running, aerobic training, and fitness room training. The total time spent in MVPA was calculated as the sum of these five activities.

Eating behavior was assessed using the Israeli Mediterranean diet screener (I-MEDAS). The I-MEDAS was adapted by a professional committee of nutritional policymakers, dieticians, and researchers to reflect the local Mediterranean diet and national dietary recommendations. The scale comprises 17 items referring to daily, weekly, and general food and drink consumption and preferences. Each item is dichotomized into 0 (not meeting the nutritional recommendations) and 1 (meeting the nutritional recommendations). Nutrition score ranges from 0 to 17, with higher scores reflecting better nutrition. The I-MEDAS has predictive utility for mortality in a population-based cohort of adults [27].

In order to assess food and drink purchases in the prison store, the participants were given a list of the different foods and drinks available in the store and asked to list three products that they usually buy. In the next step, purchases were grouped into five food and drink categories: fruits and vegetables, legumes and grains, sweets, animal products (meat, poultry, dairy), and oils and nuts. The percentage of participants buying each food and drink type was calculated.

*Emotional status.* Emotional status was assessed using seven questions pertaining to participants’ sense of anxiety, depression, serenity, energy, nervousness, happiness, and fatigue. In each question, participants were asked to rank their general feeling on a scale ranging from 1 (all the time) to 6 (never). Higher scores reflected better emotional status.

*Weight status.* The calculation of body mass index (BMI) was based on participants’ self-report of weight and height. BMI was calculated by a person’s weight in kilograms divided by their height in meters squared. Participants were then grouped into four weight groups according to their BMI: underweight (BMI < 18.5), healthy weight (BMI = 18.5–24.9), overweight (BMI = 25–29.9), or obese (BMI ≥ 30) [22]. In the regression analysis, participants were grouped into healthy and unhealthy weights (underweight, overweight, or obese). Data regarding the population’s weight status (underweight, healthy weight, overweight, or obese) was retrieved from the Israeli Ministry of Health—Centers for Disease Control and Prevention (2019).

Participants were also asked to report their body weight and height at age 18 to calculate their BMI at that time.

### 2.5. Data Management and Analysis

An assessment of the normality of the continuous data was conducted using a Kolmogorov–Smirnov test. This test is commonly used for *n* ≥ 50. The test’s null hypothesis stated that data are taken from the normal distributed population. In the current study, the *p* of all continuous variables was >0.05. Therefore, the null hypothesis is accepted, and data are deemed normally distributed.

### 2.6. Sociodemographic and Imprisonment Characteristics

Descriptive statistics (means, standard deviations, ranges, and percentages) for the sociodemographic and imprisonment characteristics were calculated.

### 2.7. Weight Change in Prison and SHS

The percentage of participants reporting gaining, losing, or no change in weight was calculated and compared using chi-square tests. Similarly, each of the five health status categories (excellent, very good, good, bad, very bad) was calculated and compared using chi-square tests.

### 2.8. Participants’ Weight Status

The prevalence of underweight, healthy weight, overweight, and obesity among study participants was calculated and compared to the population norms reported by the Israeli Ministry of Health—Centers for Disease Control and Prevention (2019) using chi-square tests. In the latter report, data were calculated according the following age groups: 18–34 years old, 35–44 years old, 45–64 years old, and ≥65 years old. In order to be able to compare the current study’s results to these of the Israeli population, similar age groups were created.

#### 2.8.1. Differences in Weight Change Based on Environmental, Emotional, Imprisonment, Socio-Demographic, and Weight Characteristics

Differences between participants with different weight change statuses (gained, lost, no change) were compared using a one-way analysis of variance (continuous variables) or chi-square tests (categorical variables). Similarly, differences between participants with different SHSs (good-to-excellent vs. bad and very bad) were compared using independent t-tests (continuous variables) or chi-square tests (categorical variables).

#### 2.8.2. Prediction of Weight Change in Prison and SHS

Variables that significantly differed statistically between the three groups of weight change or between the two groups of SHS were further analyzed using two separate binary logistic regression models to determine the extent to which the independent variables predicted weight change or SHS. In that respect, the dependent variables (weight change and SHS) were recoded as dummy variables (weight change: weight loss or no weight change = 0; weight gain = 1; SHS: bad and very bad = 0, good to excellent = 1).

Data were analyzed using the IBM SPSS statistics 25. In all statistical analyses, *p*-values < 0.05 were considered statistically significant.

### 2.9. Power Analysis

Post hoc power analysis for the multiple regression analysis was conducted using G*power version 3.1.9.4. For the first dependent variable (weight change), the effect size was calculated based on five predictors’ correlations with the dependent variable, which ranged from r = 0.005 (time in prison) to r = 0.720 (emotional status). Based on these correlations, the received p^2^ was 0.56 and the effect size f^2^ was 1.32. Based on the metrics of the five predictors included in the regression model, the study’s post hoc power was 0.99. For the second dependent variable (SHS), the effect size was also calculated based on predictors’ correlations with the dependent variable, which ranged from r = 0.001 (current BMI) to r = 0.344 (mood). Based on these correlations, the received p^2^ was 0.19 and the effect size f^2^ was 0.23. Based on the metrics with five predictors included in the regression model, the study’s post hoc power was 0.98.

## 3. Results

### 3.1. Sociodemographic and Imprisonment Characteristics

Our study included 176 male prisoners with a mean age of 39.03 + 13.99 years.

Some 50% (*n* = 89) of the prisoners were in detention for at least 5 years, while 35% and 13.6% were imprisoned for 1–5 and up to 1 year, respectively. Almost 64% of prisoners had 1–4 family visits per month, and 13% had over 4 visits a month. The majority of prisoners were either single (32%) or married (36%). Some 75% percent of prisoners were Israeli-born. The mean educational background was almost 10 years (Table 1).

### 3.2. Eating Behavior Score

No statistical differences in eating behavior scores were observed between prisoners who gained weight, lost weight, or presented no weight change (mean scores = 4.76 + 2.43, 4.61 + 2.38, and 4.57 + 2.79, respectively; F-ratio = 0.06, *p* = 0.94; Table 2).

### 3.3. Weight Status

The prevalence of participants gaining weight was significantly higher than the prevalence of those losing weight (58% and 24.4%, respectively; chi-square = 40.89, *p* < 0.001) and those with no weight change (17.6%; chi-square = 60.91, *p* < 0.001). No statistically significant differences were found in the prevalence of those losing weight and no weight change (chi-square test = 2.44, *p* = 0.11; Table 2).

**Figure 1:**
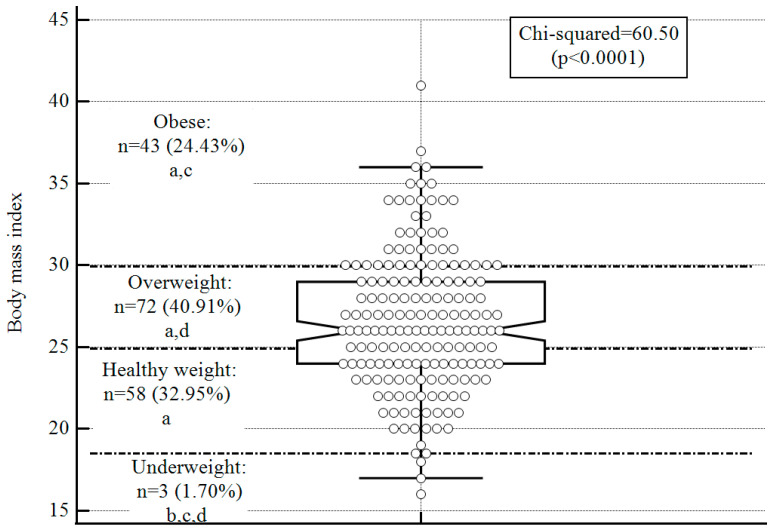
BMI of study participants (*n* = 176). Notes: a. prevalence is significantly different from “underweight” (*p* < 0.05, 2-tailed); b. prevalence is significantly different from “healthy weight” (*p* < 0.05, 2-tailed); c. prevalence is significantly different from “overweight” (*p* < 0.05, 2-tailed); d. prevalence is significantly different from “obese” (*p* < 0.05, 2-tailed); The central box represents the values from the lower to upper quartile (25–75 percentile); the vertical line extends from the minimum to the maximum value, excluding outside values. An outside value is defined as a value that is smaller than the lower quartile minus 1.5 times the interquartile range or larger than the upper quartile plus 1.5 times the interquartile range; the middle line represents the median.

**Figure 2:**
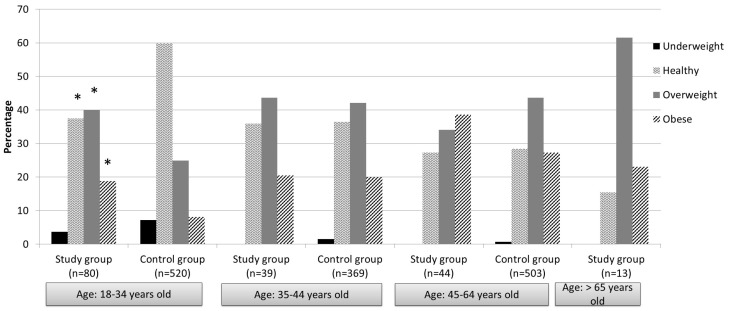
Weight status of participants (study group) and the Israeli population (control group) according to age. Notes: * Prevalence is statistically significant compared to control group (*p* < 0.0001); control group data were derived from the Israeli Ministry of Health—Centers for Disease Control and Prevention (2019).

According to participants’ self-reported current weight and height, the BMI and prevalence of overweight and obesity were calculated. Overall, the BMI range was wide, ranging from 16 to 41. The mean BMI was 26.52 + 4.29, with 65.34% defined as overweight or obese (Figure 1). In comparing participants’ BMI to that of the Israeli population, there was only a significant difference in the 18–34-year age group. More specifically, the prevalence of participants aged 18–34 with a healthy weight was significantly lower than documented in the Israeli population (37.5% and 59.8%, respectively; *p* < 0.0001). In contrast, the prevalence of overweight and obesity among the participants aged 18–34 years was significantly higher than in the Israeli population (overweight: study group: 40%, Israeli population: 24.9%, *p* < 0.0001; obese: study group: 18.8%, population: 8.1%, *p* < 0.0001; Figure 2).

#### Differences in Weight Change based on Environmental, Emotional, Imprisonment, Socio-Demographic, and Weight Characteristics

Only imprisonment duration differed between participants with various weight change statuses. More specifically, among participants who gained weight, the prevalence of those detained for at least five years (58.8%), was significantly higher than in those detained for one to five years (37.2%) or up to one year (5.8%). In contrast, the prevalence of participants who lost weight was significantly higher in those recently detained (up to one year; 30.2%). Similarly, the prevalence of reporting healthy weight was significantly higher in the group of those who reported losing weight (46.5%) than in the group of those reporting gaining weight (24.51%) or no weight change (12.7%; chi-square = 5.63, *p* = 0.01; Table 2).

### 3.4. Subjective Health Status (SHS)

Regarding SHS, the prevalence of participants who reported their health status as excellent, very good, good, bad, and very bad were: 28.9%, 19.3%, 25.0%, 18.1%, and 8.5%, respectively. The latter prevalence (very bad) was significantly lower than observed in the other four health status categories (*p* < 0.0001).

#### Differences in based on Environmental, Emotional, Imprisonment, Socio-Demographic, and Weight Characteristics

In the following analyses, participants were divided into two groups based on their SHS: those with “good-to-excellent” SHS (*n* = 129) and participants with “bad and very bad” SHS (*n* = 47). Unlike body weight change, all factors studied (except for environmental factors) differed between participants with “good-to-excellent” and “bad and very bad” subjective health. More specifically, in comparison to “bad and very bad” health, participants with “good-to-excellent” health had better emotional status, had a lower prevalence of very long-time detention (at least five years), were younger, had a higher percentage of participants with healthy current BMI, and a lower BMI at age 18 (Table 3).

### 3.5. Prediction of Weight Change in Prison and SHS

Time in prison and current BMI significantly predicted weight change. More specifically, short detention periods (up to one year) predicted less weight gain (beta coefficient = −1.69; *p* = 0.001). In contrast, current higher BMI predicted higher weight gain (beta coefficient = 0.17; *p* = 0.004). Overall, the model explained 22% of the variability observed in weight gain (χ2 = 28.55; *p* < 0.001; Table 4).

Better SHS was significantly predicted by better emotional status (beta coefficient = 0.16; *p* = 0.00013). Older age significantly predicted poorer health status (betta coefficient = −0.04; *p* = 0.0003). Overall, the model explained 22% of the variability observed in weight gain (χ^2^ = 26.25; *p* = 0.0002; Table 4).

## 4. Discussion

Our research findings demonstrate that the majority of prisoners gain weight in prison. Compared to the general population, when matched by age, prisoners gained more weight. The prevalence of overweight and obesity among participants aged 18–34 was significantly higher than in the reference Israeli population.

Two meta-analyses regarding weight change in prison found inconsistent results; Herbert et al.’s [28] analysis of 31 studies, including more than 60,000 prisoners in 884 institutions in 15 countries, found that male prisoners were less likely to be obese than males in the general population (prevalence ratios ranged from 0.33 to 0.87); however, heterogeneity in the results was noted between countries. Gebremariam et al.’s [29] analysis of 16 studies conducted in developed countries reported significant increases in body weight, BMI, or weight gain of participants. A systematic review covering 24,311 male prisoners (16–81 years old) found an obesity prevalence of 8%–56% [30]. The inconsistency regarding weight change during incarceration could be due to several reasons, including the mix of countries that were included in the analyses, with a range of low-income, middle-income, and high-income countries, the origin of the primary studies, the methodology, the varied sample sizes, the length of incarceration, and differences in prison systems. To the best of our knowledge, our findings regarding inmates’ BMI categories and changes in BMI during incarceration in Israel are novel. Israel is considered a high-income country by the World Bank [31]. Similar (but not necessarily consistent) trends were noted when examining the limited data published in similar high-income countries such as the United States [32,33].

Length of imprisonment was found to be a significant factor in weight change in prison. While an increase in weight was reported across all incarceration duration categories, inmates imprisoned for over five years gained significantly more weight than those imprisoned for shorter periods. The only group that lost weight were inmates who were in prison for less than six months. Furthermore, using multiple binary logistic regression analysis for the prediction of weight gain in prison, we found that time in prison and current weight were significant predictors of weight gain in prison. Thus, our results tentatively indicate that weight elevation is significantly linked to longer incarceration and should be addressed as early as possible, with, for example, early intervention programs. Only a few studies have assessed the relationship between imprisonment duration and weight changes, but with inconsistent results. Bailey et al. [34] did not find an overall association between the duration of incarceration and adult weight gain among men or women in the United States. Gates and Bradford [35] found that both men and women gain weight in prison, with individuals with shorter imprisonment duration gaining 2.2 times more than those with longer sentences. However, they found no relationship between being classified as overweight or obese and the duration of incarceration. Given the differences in methodology, location, and prison culture, weight gain in prison should be addressed in both a general and location-dependent manner. Our results suggest that special attention and strategies regarding weight control should be developed and applied as soon as inmates are incarcerated.

As inmates also purchased food also from the prison store, we further analyzed the association between gaining weight and the purchase of food at this venue. While we did not find the types of food purchased to be associated with weight gain, our analysis showed that the most popular products were canned fish, preserved meat, and cheeses, indicating that there may be a lack of an adequate protein dish on the prison menu. Purchases of fruits and vegetables were also reported as common despite reports of eating them every day in the prison dining room. This suggests that the quantity of those items is not satisfactory. In contrast to our results, interviews with inmates at federal prisons in Canada found that the group that gained the most weight in prison was characterized by a diet lacking vegetables, grains, legumes, and dairy products. A positive association was also found between those gaining weight and not purchasing healthy foods from the prison store; on the other hand, inmates who gained less weight reported purchasing healthy foods from the store [36].

In our study, we also examined and analyzed other factors that could affect weight change in imprisonment, such as emotional status, PA, eating behavior, family visits, and sociodemographic factors (marital status, education). However, none had a significant effect on weight status. Imprisonment can affect physical and mental health through social segregation and emotional deprivation [16]. Many studies have researched mental or emotional health in relation to imprisonment; however, there are only few studies on mental or emotional health in relation to weight. Choudhry et al. [37] interviewed male prisoners in two prisons in the United Kingdom regarding their beliefs, feelings and weight in prison, and found that the emotional consequences of imprisonment adversely affect weight. Similarly, Augsburger et al. [38] recently reported an increase in the percentage of overweight female inmates during their prison duration, which could be explained by a reduction in PA, and by diet modifications, psychological distress, and other factors.

The lack of the effect of PA on weight status and particularly on weight gain is unexpected. In previous studies, PA was found to be a major contributor to weight gain and obesity in prisons [39,40]. Johanson et al. [36] found that weight gain was less severe for inmates who engaged in regular PA. However, even high levels of PA (> 60 min/day) were not sufficient to eliminate weight gain during incarceration in Canada. Similarly, in a preliminary intervention study, Johnson et al. [41] demonstrated that a PA and dietary intervention delivered by a correctional health nurse practitioner was an efficacious approach to reducing BMI and improving resilience among female prisoners. It was suggested that gaining weight is associated with increased lean body mass due to increased time devoted to PA in prison. In our questionnaire, about 50% of the prisoners reported a decrease in PA after incarceration, which therefore did not explain our results.

Well-being indicators of inmates are disproportionately low compared with the general population. Inmates experience anxiety and distress due to external and internal extreme challenges [42,43]. Many factors may contribute to the well-being of inmates, including the prison environment and climate and individual susceptibilities and settings. Interestingly, when studying inmates’ well-being in the Netherlands, van Ginneken et al. [44] found that the vast majority of variance in well-being is on the individual level, thus indicating that self-perception is a major factor in personal well-being. However, there are possible differences across countries and prison systems. Shorter times in prison, younger age, and better emotional health were significant factors defining the inmate as having a good to excellent SHS. Such a definition in the context of prevention and management programs allows us to understand that prison administration also has a crucial effect on the subjective definition in terms of stress management, for example. Better emotional status significantly predicted better SHS, while older age significantly predicted poorer SHS.

## 5. Conclusions

This study sheds light on the nutritional and health perceptions of male inmates in 11 prisons in Israel, which is a topic that has been largely overlooked in the existing research. Our findings revealed a concerning prevalence of overweight and obesity among young inmates, as well as the impact of short detention periods and age on weight gain and health status. The study emphasizes the need for nutrition interventions to improve the health of inmates and highlights the importance of promoting a healthier lifestyle in prison from the outset. Overall, this research provides valuable insights into the health and well-being of inmates and highlights the need for further research in this area to support the development of effective interventions and policies that can help to improve their overall health and well-being.

## Figures and Tables

**Table 1:** Sociodemographic and imprisonment characteristics of the sample (*n* = 176).

Variables			Mean (SD) [Range] OR *n* (%)
Sociodemographic characteristics	Age, years: mean (SD) [range]		39.03 (13.99) [18.00–82.00]
	Gender: *n* (%)	Male	176 (100.00)
	Country of origin: *n* (%)	Native to the country	133 (75.56)
		Foreign	43 (24.43)
	Marital status: *n* (%)	Single	56 (31.82)
		In partnership	14 (7.95)
		Married	61 (34.66)
		Separated	9 (5.11)
		Divorced	28 (15.91)
		Widowed	8 (4.55)
	School, years: mean (SD) [range]		9.88 (3.24) [0.00–15.00]
	Educational level: *n* (%)	Never went to school	11 (6.25)
		Only primary school	36 (20.45)
		Graduated from school without a diploma	33 (18.75)
		Basic diploma	41 (23.30)
		Professional diploma	23 (13.07)
		College diploma	7 (3.98)
		Technical or professional certification	9 (5.11)
		Professional degree/course	16 (9.09)
Imprisonment characteristics	Years of detention: *n* (%)	Up to one year: *n* (%)	24 (13.64)
		One to five years: *n* (%)	63 (35.80)
		At least five years: *n* (%)	89 (50.57)
	Family visits in the previous month: *n* (%)	Never	41 (23.30)
		Once	57 (32.39)
		Two to four	55 (31.25)
		More than four	23 (13.07)

**Table 2:** Differences in weight change based on environmental, emotional, imprisonment, sociodemographic, and weight characteristics (*n* = 176).

Independent Variables			Weight Change			Between Group Comparison
			Gained Weight: Mean (SD) OR *n* (%)	Lost Weight: Mean (SD) OR *n* (%)	No Weight Change: Mean (SD)_ OR *n* (%)	F-Ratio (*p*-Value) OR Chi-Square (*p*-Value)
Total: *n* (%)			102 (57.95)	43 (24.43)	31 (17.61)	50.90 (0.001)
Environmental factors	Physical activity level, minutes: mean (SD)		115.49 (242.20)	74.79 (103.75)	115.48 (203.20)	1.06 (0.70)
	Eating behavior, score: mean (SD)		4.76 (2.43)	4.61 (2.38)	4.57 (2.79)	0.06 (0.94)
	Products bought in the prison store	Fruits/vegetables, yes: *n* (%)	8 (7.84)	5 (11.63)	3 (9.68)	0.60 (0.73)
		Legumes/grains, yes: *n* (%)	47 (46.07)	19 (44.19)	11 (35.45)	1.08 (0.58)
		Sweets, yes: *n* (%)	32 (31.37)	12 (27.91)	11 (35.45)	0.42 (0.80)
		Animal products, yes: *n* (%)	62 (60.78)	26 (60.46)	21 (67.74)	0.50 (0.77)
		Oils/nuts, yes: *n* (%)	24 (23.53)	4 (9.30)	5 (16.13)	4.18 (0.12)
Emotional status, score: mean (SD)			20.74 (4.78)	20.92 (4.74)	21.06 (4.26)	0.06 (0.94)
Imprisonment factors	Prison time	Up to one year: *n* (%)	6 (5.88) ^b,c^	13 (30.23) ^a,c^	5 (16.12) ^a,b^	16.35 (0.002)
		One to five years: *n* (%)	38 (37.25)	12 (27.90) ^c^	13 (41.94) ^b^	12.35 (0.003)
		At least five years: *n* (%)	58 (58.86) ^b,c^	18 (41.86) ^a^	13 (41.94) ^a^	16.46 (0.002)
	Family visits	No: *n* (%)	26 (25.49)	6 (13.95)	9 (29.03)	2.96 (0.22)
Sociodemographic factors	Age, years: mean (SD)		39.13 (13.96)	39.08 (15.60)	38.60 (12.01)	0.01 (0.98)
	Native to the country, yes: *n* (%)		79 (77.45)	32 (74.42)	22 (70.97)	0.58 (0.74)
	Marital status, in a relationship: *n* (%)		37 (36.27)	24 (55.81)	14 (45.16)	4.82 (0.08)
	Education, school years: mean (SD)		9.67 (3.50)	10.51 (2.13)	9.76 (3.53)	3.56 (0.03)
Weight status	Current BMI: mean (SD)		27.63 (4.32) ^b^	24.53 (3.54) ^a^	25.64 (4.00)	9.55 (<0.001)
	Current BMI status, healthy weight: *n* (%)		25 (24.51) ^b,c^	20 (46.51) ^a,c^	13 (12.75)^a,b^	5.63 (0.01)
	BMI at 18: mean (SD)		20.23 (7.54)	17.44 (10.65)	17.74 (9.35)	2.01 (0.13)

Notes: ^a^. statistically significantly different from the “gained weight” group (*p* < 0.05; 2-tailed); ^b^. statistically significantly different from the “lost weight” group (*p* < 0.05; 2-taled); ^c^. statistically significantly different from the “no weight change” group (*p* < 0.05; 2-taled); SD, standard deviation.

**Table 3:** Differences in SHS based on environmental, emotional, imprisonment, sociodemographic, and weight characteristics (*n* = 176).

Independent Variables				SHS		Between-Group Comparison
				Good, Very Good, Excellent (*n* = 129): Mean (SD) OR *n* (%)	Bad and Very Bad (*n* = 47): Mean (SD) OR *n* (%)	Statistic t (*p*-Value) OR Chi-Square (*p*-Value)
Environmental factors	PA level, minutes: mean (SD)			129.81 (290.25)	121.25 (330.21)	−0.16 (0.86)
	Eating behavior, score: mean (SD)			4.82 (2.41)	4.31 (2.49)	−0.96 (0.33)
	Products bought in the prison store	Fruits/vegetables, yes: *n* (%)		13 (10.07)	3 (6.38)	0.56 (0.45)
		Legumes/grains, yes: *n* (%)		54 (41.86)	23 (48.94)	0.69 (0.40)
		Sweets, yes: *n* (%)		42 (32.56)	13 (27.66)	0.38 (0.53)
		Animal products, yes: *n* (%)		80 (62.02)	29 (61.70)	0.01 (0.96)
		Oils/nuts, yes: *n* (%)		25 (19.38)	8 (17.02)	0.12 (0.72)
Emotional status, score: mean (SD)				21.52 (4.46)	18.88 (4.70)	−3.29 (0.001)
Imprisonment factors	Prison time		Up to one year: *n* (%)	19 (14.73)	5 (10.64)	0.48 (0.48)
			One to five years: *n* (%)	52 (40.31)	11 (23.40)	5.96 (0.01)
			At least five years: *n* (%)	58 (44.96)	31 (65.95)	6.01 (0.01)
	Family visits		No: *n* (%)	102 (79.07)	33 (70.21)	1.50 (0.22)
Sociodemographic factors	Age, years: mean (SD)			37.41 (13.02)	43.46 (15.66)	2.58 (0.01)
	Native to the country, yes: *n* (%)			97 (75.19)	36 (76.60)	0.03 (0.84)
	Marital status, in a relationship: *n* (%)			57 (44.19)	18 (38.30)	0.48 (0.48)
	Education: school years: mean (SD)			9.68 (3.25)	10.45 (3.19)	1.28 (0.20)
Weight status	Current BMI: mean (SD)			22.39 (1.60)	15.28 (<0.0001)	15.28 (<0.001)
	Current BMI status, healthy weight: *n* (%)			45 (34.89)	13 (27.66)	0.36 (0.06)
	BMI at 18: mean (SD)			16.08 (9.15)	20.60 (8.21)	3.18 (0.001)

**Table 4:** Summary of multiple binary logistic regression analysis for prediction of weight gain in prison.

Dependent Variables	Independent Variables		Coefficient	Standard Error	Odds Ratio	Wald	95% Confidence Interval	*p*
Weight change (reference—gained weight)	Constant		−4.17	1.76	-	5.56	-	0.01
	Time in prison (reference value—at least five years)	Up to one year	−1.69	0.54	0.18	9.63	0.06–0.53	0.001
		One to five years	−0.19	0.35	0.82	0.29	0.40–1.66	0.58
	Current BMI		0.17	0.06	1.19	8.10	1.05–1.35	0.004
	BMI category	Healthy	0.22	0.49	1.25	0.20	0.47–3.32	0.64
	*Model summary*		Chi-Square = 28.55, *p* < 0.0001, Nagelkerke R^2^ = 0.22					
SHS: reference: bad and very bad)	Constant		−0.67	1.60	-	0.17	-	0.67
	Emotional status score		0.16	0.04	1.17	12.78	1.07–1.28	0.0003
	Time in prison	Up to one year	0.32	0.59	1.38	0.30	0.43–4.44	0.58
		One to five years	0.83	0.45	2.31	3.44	0.95–5.61	0.06
	Age, years		−0.04	0.01	0.95	8.50	0.93–0.98	0.0003
	Current BMI		0.01	0.04	1.01	0.04	0.92–1.11	0.82
	BMI at 18		−0.01	0.02	0.98	0.17	0.93–1.02	0.40
	*Model summary*		Chi-square = 26.25, *p* = 0.0002, Nagelkerke R^2^ =0.22					

Notes: Only variables that significantly (*p* < 0.05; 2-tailed) differed between participants with different weights and SHS were included in the regression model.

## Data Availability

Data are unavailable due to privacy or ethical restrictions.
